# 4-Meth­oxy-*N*-phenyl­aniline

**DOI:** 10.1107/S1600536808039457

**Published:** 2008-12-17

**Authors:** Karol Krzymiński, Michał Wera, Artur Sikorski, Jerzy Błażejowski

**Affiliations:** a Faculty of Chemistry, University of Gdańsk, J. Sobieskiego 18, 80-952 Gdańsk, Poland

## Abstract

In the mol­ecule of the title compound, C_13_H_13_NO, the two benzene rings are oriented at a dihedral angle of 59.9 (2)°. In the crystal structure, the benzene rings of neighbouring mol­ecules are oriented nearly parallel or perpendicular, making dihedral angles of 2.8 (2) and 79.5 (2)°, respectively. The crystal structure is stabilized by a network of C—H⋯π and N—H⋯π inter­actions.

## Related literature

For general background, see: Acheson (1973 ▶); Gatto *et al.* (2006 ▶); Li *et al.* (2002 ▶); Oettmeier & Renger (1980 ▶); Razavi & McCapra (2000**a* ▶,b* ▶); Steiner (2000 ▶); Takahashi *et al.* (2001 ▶); Velusamy *et al.* (2005 ▶); Zomer & Jacquemijns (2001 ▶). For related structures, see: Rodriguez & Bunge (2003 ▶).
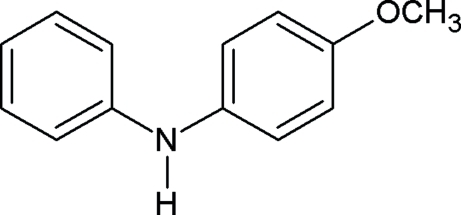


## Experimental

### 

#### Crystal data

C_13_H_13_NO
*M* *_r_* = 199.24
Orthorhombic, 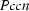

*a* = 15.090 (3) Å
*b* = 18.394 (4) Å
*c* = 7.596 (2) Å
*V* = 2108.4 (8) Å^3^
*Z* = 8
Mo *K*α radiation
μ = 0.08 mm^−1^
*T* = 295 (2) K
0.05 × 0.03 × 0.02 mm

#### Data collection

Kuma KM-4 diffractometer
Absorption correction: none
2443 measured reflections
1851 independent reflections
1005 reflections with *I* > 2σ(*I*)
*R*_int_ = 0.035
3 standard reflections every 200 reflections intensity decay: 0.5%

#### Refinement

*R*[*F* ^2^ > 2σ(*F* ^2^)] = 0.044
*wR*(*F* ^2^) = 0.131
*S* = 0.99
1851 reflections
138 parameters
H-atom parameters constrained
Δρ_max_ = 0.16 e Å^−3^
Δρ_min_ = −0.15 e Å^−3^

### 

Data collection: *KM-4 Software* (Oxford Diffraction, 2003 ▶); cell refinement: *KM-4 Software*; data reduction: *KM-4 Software*; program(s) used to solve structure: *SHELXS97* (Sheldrick, 2008 ▶); program(s) used to refine structure: *SHELXL97* (Sheldrick, 2008 ▶); molecular graphics: *ORTEPII* (Johnson, 1976 ▶); software used to prepare material for publication: *SHELXL97* and *PLATON* (Spek, 2003 ▶).

## Supplementary Material

Crystal structure: contains datablocks global, I. DOI: 10.1107/S1600536808039457/hk2570sup1.cif

Structure factors: contains datablocks I. DOI: 10.1107/S1600536808039457/hk2570Isup2.hkl

Additional supplementary materials:  crystallographic information; 3D view; checkCIF report

## Figures and Tables

**Table 1:** C—H⋯π and N—H⋯π inter­actions (Å, °)

*D*—H⋯*A*	*D*—H	H⋯*A*	*D*⋯*A*	*D*—H⋯*A*
C3—H3⋯*Cg*2^i^	0.93	2.91	3.671 (2)	139
N7—H7⋯*Cg*1^ii^	0.86	2.88	3.593 (2)	142
C10—H10⋯*Cg*1^iii^	0.93	2.92	3.723 (3)	145

Symmetry codes: (i) 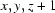; (ii) 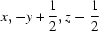; (iii) 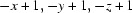. *Cg*1 and *Cg*2 are the centroids of the C1–C6 ring and C8–C13 rings, respectively.

**Fig. 1.**
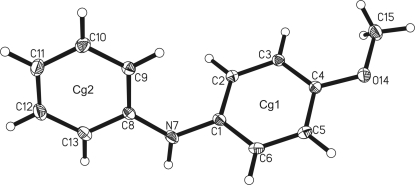
The molecular structure of the title molecule, with the atom-numbering scheme. Displacement ellipsoids are drawn at the 25% probability level. Cg1 and Cg2 denote the ring centroids.

**Fig. 2.**
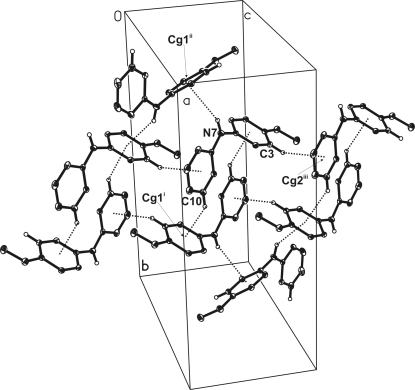
The arrangement of the molecules in the crystal packing, viewed approximately along the a axis. The C—H···π and N—H···π interactions are represented by dotted lines. H atoms not involved in interactions have been omitted [symmetry codes: (i) x, y, 1 + z; (ii) x, 1/2 - y, -1/2 + z; (iii) 1 - x, 1 - y, 1 - z].

## supplementary crystallographic information

### Comment

Diphenylamines are an important class of aromatic amines widely employed in organic (Acheson, 1973) and organometallic (Li *et al.*, 2002) syntheses. They exhibit interesting biological activities (Oettmeier & Renger, 1980). Some of them are known to be useful antioxidants for modern lubricants (Gatto *et al.*, 2006) or as fragments of molecules with interesting electro-optical properties (Velusamy *et al.*, 2005). Diphenylamines are precursors of acridine-9-carboxylic acids (Acheson, 1973; Zomer & Jacquemijns, 2001), which are starting materials for syntheses of acridinium chemiluminogenic tracers (Razavi & McCapra, 2000*a*,b; Zomer & Jacquemijns, 2001). The presence of a methoxy group in such tracers enhances their stability in an aquatic environment and brings about a red shifting of the emitted light. The latter feature should increase the potential applicability of acridinium chemiluminogens in immunoassays (Zomer & Jacquemijns, 2001).

In the molecule of the title compound (Fig. 1) the bond lengths and angles are in accordance with the corresponding values in diphenylamine (Rodriguez & Bunge, 2003). Rings A (C1-C6) and B (C8-C13) are planar and oriented at a dihedral angle of 59.9 (2)°.

In the crystal structure, benzene ring systems of neighbouring molecules are oriented nearly parallel or perpendicular. The respective angles between them are 2.8 (2)° and 79.5 (2)°. The crystal structure of the title compound is stabilized by a network of specific C—H···π and N—H···π interactions (Fig. 2 and Table 1) which exhibit an attractive nature (Steiner, 2000; Takahashi *et al.*, 2001), as well as by non-specific dispersive interactions.

### Experimental

The title compound was synthesized by the condensation of 4-methoxy-benzenamine and bromobenzene in the presence of anhydrous potassium carbonate and a catalytic amount of copper iodide (yield; 75%) (Zomer & Jacquemijns, 2001). Elemental analysis (% found/calculated): C 78.16/78.36, H 6.58/6.72, N 7.02/7.03. Colorless crystals (m.p. 379-380 K) suitable for X-ray analysis were grown from absolute ethanol solution.

### Refinement

H atoms were positioned geometrically, with N-H = 0.86 Å and C-H = 0.93 and 0.96 Å for aromatic and methyl H, respectively, and constrained to ride on their parent atoms with U_iso_(H) = xU_eq_(C,N), where x = 1.5 for methyl H and x = 1.2 for all other H atoms.

### Crystal data

**Table:**

C_13_H_13_NO	*F*(000) = 848
*M**_r_* = 199.24	*D*_x_ = 1.255 Mg m^−^^3^
Orthorhombic, *P**c**c**n*	Mo *K*α radiation, λ = 0.71073 Å
Hall symbol: -P 2ab 2ac	Cell parameters from 50 reflections
*a* = 15.090 (3) Å	θ = 2.2–25°
*b* = 18.394 (4) Å	µ = 0.08 mm^−^^1^
*c* = 7.596 (2) Å	*T* = 295 K
*V* = 2108.4 (8) Å^3^	Block, colorless
*Z* = 8	0.05 × 0.03 × 0.02 mm

### Data collection

**Table:**

Kuma KM-4 diffractometer	*R*_int_ = 0.035
Radiation source: fine-focus sealed tube	θ_max_ = 25.0°, θ_min_ = 2.2°
graphite	*h* = 0→17
θ/2θ scans	*k* = −21→0
2443 measured reflections	*l* = −9→2
1851 independent reflections	3 standard reflections every 200 reflections
1005 reflections with *I* > 2σ(*I*)	intensity decay: 0.5%

### Refinement

**Table:**

Refinement on *F*^2^	Secondary atom site location: difference Fourier map
Least-squares matrix: full	Hydrogen site location: inferred from neighbouring sites
*R*[*F*^2^ > 2σ(*F*^2^)] = 0.044	H-atom parameters constrained
*wR*(*F*^2^) = 0.131	*w* = 1/[σ^2^(*F*_o_^2^) + (0.081*P*)^2^ + 0.0542*P*] where *P* = (*F*_o_^2^ + 2*F*_c_^2^)/3
*S* = 0.99	(Δ/σ)_max_ < 0.001
1851 reflections	Δρ_max_ = 0.16 e Å^−^^3^
138 parameters	Δρ_min_ = −0.15 e Å^−^^3^
0 restraints	Extinction correction: *SHELXL97* (Sheldrick, 2008), Fc^*^=kFc[1+0.001xFc^2^λ^3^/sin(2θ)]^-1/4^
Primary atom site location: structure-invariant direct methods	Extinction coefficient: 0.015 (2)

### Special details

**Table:**

Geometry. All e.s.d.'s (except the e.s.d. in the dihedral angle between two l.s. planes) are estimated using the full covariance matrix. The cell e.s.d.'s are taken into account individually in the estimation of e.s.d.'s in distances, angles and torsion angles; correlations between e.s.d.'s in cell parameters are only used when they are defined by crystal symmetry. An approximate (isotropic) treatment of cell e.s.d.'s is used for estimating e.s.d.'s involving l.s. planes.

### Fractional atomic coordinates and isotropic or equivalent isotropic displacement parameters (Å^2^)

**Table:**

	*x*	*y*	*z*	*U*_iso_*/*U*_eq_	
C1	0.49051 (14)	0.32843 (9)	0.6655 (3)	0.0526 (6)	
C2	0.52660 (14)	0.36650 (10)	0.8048 (3)	0.0533 (5)	
H2	0.5861	0.3794	0.8010	0.064*	
C3	0.47688 (12)	0.38576 (10)	0.9489 (3)	0.0500 (5)	
H3	0.5025	0.4117	1.0409	0.060*	
C4	0.38875 (13)	0.36634 (10)	0.9558 (3)	0.0491 (5)	
C5	0.35215 (14)	0.32732 (10)	0.8191 (3)	0.0553 (6)	
H5	0.2930	0.3133	0.8244	0.066*	
C6	0.40192 (15)	0.30921 (10)	0.6763 (3)	0.0577 (6)	
H6	0.3760	0.2835	0.5843	0.069*	
N7	0.54179 (14)	0.30655 (9)	0.5208 (2)	0.0679 (6)	
H7	0.5415	0.2610	0.4954	0.082*	
C8	0.59269 (13)	0.35133 (11)	0.4155 (3)	0.0488 (5)	
C9	0.59081 (13)	0.42662 (10)	0.4292 (3)	0.0535 (5)	
H9	0.5561	0.4486	0.5153	0.064*	
C10	0.63953 (16)	0.46850 (13)	0.3170 (3)	0.0701 (7)	
H10	0.6377	0.5188	0.3279	0.084*	
C11	0.69087 (19)	0.43785 (18)	0.1892 (3)	0.0882 (9)	
H11	0.7233	0.4669	0.1124	0.106*	
C12	0.69399 (16)	0.36363 (17)	0.1759 (3)	0.0809 (8)	
H12	0.7291	0.3422	0.0897	0.097*	
C13	0.64619 (14)	0.32079 (13)	0.2877 (3)	0.0633 (6)	
H13	0.6497	0.2705	0.2777	0.076*	
O14	0.33254 (9)	0.38212 (8)	1.0914 (2)	0.0671 (5)	
C15	0.36725 (18)	0.41960 (15)	1.2379 (3)	0.0809 (7)	
H15A	0.3220	0.4246	1.3260	0.121*	
H15B	0.3871	0.4669	1.2018	0.121*	
H15C	0.4162	0.3928	1.2857	0.121*	

### Atomic displacement parameters (Å^2^)

**Table:**

	*U*^11^	*U*^22^	*U*^33^	*U*^12^	*U*^13^	*U*^23^
C1	0.0758 (14)	0.0385 (10)	0.0434 (11)	−0.0006 (9)	0.0030 (11)	0.0020 (9)
C2	0.0537 (11)	0.0521 (11)	0.0542 (13)	−0.0044 (9)	0.0006 (10)	−0.0014 (9)
C3	0.0555 (11)	0.0497 (10)	0.0448 (11)	−0.0027 (9)	−0.0027 (10)	−0.0019 (9)
C4	0.0534 (11)	0.0462 (10)	0.0475 (11)	0.0004 (9)	0.0005 (10)	0.0042 (9)
C5	0.0531 (12)	0.0536 (11)	0.0593 (14)	−0.0081 (9)	−0.0085 (11)	0.0039 (10)
C6	0.0764 (15)	0.0458 (11)	0.0510 (13)	−0.0124 (10)	−0.0146 (11)	0.0016 (10)
N7	0.1075 (14)	0.0417 (8)	0.0546 (11)	−0.0010 (9)	0.0181 (11)	−0.0072 (8)
C8	0.0529 (11)	0.0553 (11)	0.0383 (10)	0.0050 (8)	−0.0078 (9)	−0.0012 (9)
C9	0.0610 (12)	0.0523 (11)	0.0471 (12)	0.0029 (9)	−0.0039 (10)	0.0022 (10)
C10	0.0821 (16)	0.0706 (14)	0.0577 (15)	−0.0181 (12)	−0.0034 (13)	0.0091 (12)
C11	0.0803 (17)	0.125 (2)	0.0595 (17)	−0.0421 (17)	0.0081 (14)	−0.0006 (16)
C12	0.0543 (13)	0.126 (2)	0.0622 (16)	−0.0077 (14)	0.0080 (12)	−0.0266 (15)
C13	0.0589 (13)	0.0755 (14)	0.0556 (13)	0.0104 (11)	−0.0050 (11)	−0.0181 (12)
O14	0.0578 (9)	0.0794 (10)	0.0642 (10)	−0.0026 (7)	0.0064 (7)	−0.0103 (8)
C15	0.0766 (15)	0.1028 (18)	0.0634 (16)	0.0028 (14)	0.0076 (14)	−0.0203 (15)

### Geometric parameters (Å, °)

**Table:**

C1—C2	1.381 (3)	C8—C9	1.389 (3)
C1—C6	1.385 (3)	C9—C10	1.364 (3)
C1—N7	1.403 (3)	C9—H9	0.9300
C2—C3	1.373 (3)	C10—C11	1.364 (4)
C2—H2	0.9300	C10—H10	0.9300
C3—C4	1.378 (3)	C11—C12	1.370 (4)
C3—H3	0.9300	C11—H11	0.9300
C4—O14	1.365 (2)	C12—C13	1.365 (3)
C4—C5	1.378 (3)	C12—H12	0.9300
C5—C6	1.361 (3)	C13—H13	0.9300
C5—H5	0.9300	O14—C15	1.410 (3)
C6—H6	0.9300	C15—H15A	0.9600
N7—C8	1.381 (3)	C15—H15B	0.9600
N7—H7	0.8600	C15—H15C	0.9600
C8—C13	1.382 (3)		
			
C2—C1—C6	117.68 (19)	C13—C8—C9	118.0 (2)
C2—C1—N7	121.9 (2)	C10—C9—C8	120.4 (2)
C6—C1—N7	120.36 (19)	C10—C9—H9	119.8
C3—C2—C1	121.74 (19)	C8—C9—H9	119.8
C3—C2—H2	119.1	C9—C10—C11	121.1 (2)
C1—C2—H2	119.1	C9—C10—H10	119.4
C2—C3—C4	119.42 (19)	C11—C10—H10	119.4
C2—C3—H3	120.3	C10—C11—C12	119.0 (2)
C4—C3—H3	120.3	C10—C11—H11	120.5
O14—C4—C3	125.00 (18)	C12—C11—H11	120.5
O14—C4—C5	115.48 (17)	C13—C12—C11	120.8 (2)
C3—C4—C5	119.52 (19)	C13—C12—H12	119.6
C6—C5—C4	120.48 (18)	C11—C12—H12	119.6
C6—C5—H5	119.8	C12—C13—C8	120.7 (2)
C4—C5—H5	119.8	C12—C13—H13	119.6
C5—C6—C1	121.15 (19)	C8—C13—H13	119.6
C5—C6—H6	119.4	C4—O14—C15	117.9 (2)
C1—C6—H6	119.4	O14—C15—H15A	109.5
C1—N7—C8	126.1 (2)	O14—C15—H15B	109.5
C8—N7—H7	116.9	H15A—C15—H15B	109.5
C1—N7—H7	116.9	O14—C15—H15C	109.5
N7—C8—C13	119.30 (19)	H15A—C15—H15C	109.5
N7—C8—C9	122.67 (18)	H15B—C15—H15C	109.5
			
C6—C1—C2—C3	−0.8 (3)	C1—N7—C8—C13	174.0 (2)
N7—C1—C2—C3	−177.82 (17)	C1—N7—C8—C9	−7.8 (3)
C1—C2—C3—C4	0.4 (3)	N7—C8—C9—C10	−177.21 (19)
C2—C3—C4—O14	179.92 (17)	C13—C8—C9—C10	1.1 (3)
C2—C3—C4—C5	0.6 (3)	C8—C9—C10—C11	0.1 (3)
O14—C4—C5—C6	179.34 (17)	C9—C10—C11—C12	−0.9 (4)
C3—C4—C5—C6	−1.3 (3)	C10—C11—C12—C13	0.4 (4)
C4—C5—C6—C1	0.9 (3)	C11—C12—C13—C8	0.8 (4)
C2—C1—C6—C5	0.1 (3)	N7—C8—C13—C12	176.8 (2)
N7—C1—C6—C5	177.18 (18)	C9—C8—C13—C12	−1.5 (3)
C2—C1—N7—C8	−55.5 (3)	C3—C4—O14—C15	−1.6 (3)
C6—C1—N7—C8	127.6 (2)	C5—C4—O14—C15	177.7 (2)

### Hydrogen-bond geometry (Å, °)

**Table:**

*D*—H···*A*	*D*—H	H···*A*	*D*···*A*	*D*—H···*A*
C3—H3···Cg2^i^	0.93	2.91	3.671 (2)	139
N7—H7···Cg1^ii^	0.86	2.88	3.593 (2)	142
C10—H10···Cg1^iii^	0.93	2.92	3.723 (3)	145

Symmetry codes: (i) *x*, *y*, *z*+1; (ii) *x*, −*y*+1/2, *z*−1/2; (iii) −*x*+1, −*y*+1, −*z*+1.
